# Supplementary material for: Lung-protective properties of expiratory flow-initiated pressure-controlled inverse ratio ventilation: A randomised controlled trial
Source: PLoS One. 2020 Dec 17;15(12):e0243971. doi: 10.1371/journal.pone.0243971 (PMC7746151; doi:10.1371/journal.pone.0243971)
Supplement: S1 File — (DOCX) [file pone.0243971.s002.docx]

**研究実施計画書**

1. 課題名：換気効率が高く肺保護的な呼吸モード設定の探求
2. 研究の意義（背景）と目的

　ARDS、分離肺換気手術、気腹手術、肥満患者など、炎症あるいは肺低コンプライアンス症例では、肺胞の過膨張過進展を防ぐ目的で低一回換気量、無期肺予防としてPEEPで人工呼吸を行うことが肺保護的と考えられている。しかしそれら肺保護戦略は通常は従量式換気Volume control ventilation（VCV)で吸気・呼気比（I：E比）１：２で行われている。我々はすでに、十分な吸気プラトー時間で行う逆比従圧式換気Pressure control inverse ratio ventilation (PC-IRV)がI:E比1:2のVCVよりも死腔が少なく、換気効率が高い換気方法であることを見出した。より少ない換気量で効率よく二酸化炭素を排出できるPC-IRVは肺保護的換気法となりうると考えた。

従来肺保護的であるとされる低換気量・VCV・I:E1:2と換気効率が高いPC-IRVの肺保護性をランダム化比較試験で検討することを目的とする。

1. 研究の方法：

【研究デザイン】ランダム化比較試験

【対象】18歳以上，85歳未満のロボット支援前立腺全摘術の予定手術患者を対象とする。ASA3以上、緊急手術は除外する。

【設定】帝京大学医学部附属溝口病院手術室

【介入】無作為に呼吸モードをVCV群PC-IRV群に分類する。両群とも動脈血液ガスPaO_2_>100mmHg, PaCO_2_ 45~50mmHgを目標として、それぞれ以下の指定された戦略に従い換気設定を行う。

VCV群の換気戦略

　初期設定として、呼吸モードをVCV、最高気道内圧＜30 cmH_2_Oとなるように一回換気量TV 6-8 ml/kg(理想体重）に調節し、呼吸回数12回/min、I:E = 1:2、PEEP 5 cmH_2_O、ポーズ比2０％とする。動脈血液ガス採取時のPaCO_2_ と呼気二酸化炭素濃度E_T_CO_2_の格差をもとに、その後はE_T_CO_2_の値からPaCO_2_ を推測して、PaCO_2_ 45~50mmHgを目標に呼吸回数を12～20回/分の範囲内で調節する。最高気道内圧が高い場合はPEEPを必要に応じて低下させる。PaO_2_>100mmHgを達成することを目標にFiO_2_を設定する。呼吸回数増加によっても目標PaCO_2_ 45~50mmHg達成困難の場合はPaCO_2_増加を許容する（permissive hypercapnia)。

PC-IRV群の換気戦略

　肺過膨張を来たさず換気効率を追求したPC-IRVを行う。auto PEEPによる肺過膨張を来たさない程度の最小呼気時間となるよう、呼気フロー波形から適切な吸気開始が行われるようにI:E比を設定。換気効率が低下しないよう十分に必要吸気時間が得られる呼吸回数を設定する。初期設定として、呼吸モードを換気量をターゲットにした圧制御(PCV-VG)、最高気道内圧＜25 cmH_2_Oとなるように一回換気量TV 6-10 ml/kg(理想体重）に調節し、呼吸回数12回/min、PEEPオフとする。動脈血液ガス採取時のPaCO_2_ と呼気二酸化炭素濃度E_T_CO_2_の格差をもとに、その後はE_T_CO_2_の値からPaCO_2_ を推測して、PaCO_2_ 45~50mmHgを目標に呼吸回数を12～20回/分の範囲内で調節する。呼吸回数変更に応じて呼気フロー波形から適切な吸気開始が行われるようにI:E比を設定する。PaO_2_>100mmHgを達成することを目標にFiO_2_を設定する。

　VCV群PC-IRV群それぞれ麻酔は循環動態を安定させるために測定中は硬膜外単回投与を行わず、筋弛緩薬を持続投与する。動脈血採血と循環動態の把握のために橈骨動脈カテーテルを挿入し、フロートラックセンサを用いて循環動態を評価する。ボリュームカプノメトリを用いて死腔および二酸化炭素排出量を測定する。

【測定項目】

主要アウトカム

　手術開始後３時間あるいは手術終了時の動脈血液 IL-6（肺障害の指標）

副次的アウトカム

　手術開始後3時間あるいは手術終了時の動脈血液IL-1β、 IL-8、TNFα

　生理学的死腔換気率（換気効率の指標）

　その他、手術開始前、開始後3時間あるいは手術終了時の動脈血液ガス分析によるPaO_2_、PaCO_2_、各種換気パラメーター（一回換気量、呼吸回数、分時換気量、最高気道内圧、吸気プラトー圧）、循環パラメーター（血圧、心拍数、およびフロートラックセンサーから心係数CI、1回拍出量変化率SVV）。術中・術後の合併症。

【ランダム化】

割り振りのタイプ

　登録割付業務を中央登録割付とする。登録、割付に関しては、研究者とは独立に、登録・割付の責任者/担当者が実施する。テーブル（割付表）の作成はTARCが行い、試験開始前に登録・割付責任者/担当者に渡す。割付の方法は可変ブロックとし、ブロックサイズはTARC内にて管理する。

割付けの隠蔵

　手術開始直前に、研究者より登録・割付責任者/担当者にメールにて手術の準備が整った旨連絡し、登録・割付担当者は、割付を実施し割付結果を麻酔医に知らせる。この具体的な方法に関しては、別途手順書により定める。本ランダム化により患者選択の恣意性については最小化されることが期待できる。

【統計】

Student test あるいは Mann–Whitney U testで行う。

1. 目標対象者数：

類似研究の結果から、IL-6濃度平均値の差を70pg/ml、標準偏差 40、パワー90%、危険度5%として計算した結果、サンプルサイズはVCV群、PC-IRV群それぞれ14例を予定する。

1. 研究組織（○本学における実施責任者　◎本学における情報管理責任者　＃登録割り付け担当者）：

○◎平林　剛（帝京大学溝口病院麻酔科　講師）

　　安藤富男（帝京大学溝口病院麻酔科　教授）

＃　丸山晃一（帝京大学溝口病院麻酔科　教授）

　　秋久友希（帝京大学溝口病院麻酔科　助教）

　　西岡　浩子（帝京大学溝口病院麻酔科　助教）

　　鴨志田直子（帝京大学溝口病院麻酔科　助手）

1. 研究期間：

倫理委員会承認後～2022年12月31日まで。

1. 研究における倫理的配慮：

　本研究は、「ヘルシンキ宣言に基づく倫理的原則」および「人を対象とする医学系研究に関する倫理指針」に従い、本計画書を遵守して実施する。

1）インフォームド・コンセント

□研究の目的・意義

□研究の対象と方法

□研究への自由意思参加・同意取消しの自由

□研究の責任者・組織

□研究の場所・期間

□研究試料と情報の取り扱い

□研究結果の扱い

□研究資金源

□利益相反

□研究参加者の負担や支払いの有無

□被る可能性のある個人の利益、不利益、有害事象とその対応

□研究中止の条件

□質問への対応の仕方・連絡先

本研究実施責任者または担当者が前日までに被験者の元に行き、上記説明書を元に口頭で説明し、インフォームドコンセントを得る。インフォームドコンセントが得られない場合、研究は行わない。同意書取得の後、参加者が質問、同意の取り消しなどで連絡したい場合は病棟看護師より電話：044-844-3333内線8233麻酔科平林剛まで電話連絡してもらう。

2）情報（研究に用いられる情報に係る資料を含む）の保管及び廃棄の方法

　　「臨床研究における記録保管に関する標準業務手順書」に従う。情報の匿名化は行わない。情報の保管方法は以下のとおりである。

　記録① 倫理委員会申請書・承認書・報告書を、倫理委員会事務局内の施錠された鍵棚に保管する。ただし、研究終了から10年を経過した研究に関する原本は、記録保管責任者の判断により、外部委託先倉庫に保管することも可能とする。

　記録② 電子カルテ上の診療情報電子はカルテサーバーに保管される。

　記録③　上記以外の記録に関しては、研究終了前は研究責任者が保管の対象となる記録類一式を講座内の施錠できるキャビネットに保管する。記録保管責任者は、記録類一式が適切に保管されるよう必要な監督を行う。研究終了後はデータセット等を倫理委員会事務局に提出し、TARCにて10年間保管の後に廃棄する。

　情報の取り扱い者及び責任者；平林剛　（帝京大学溝口病院麻酔科　講師）

　問い合わせ・苦情に対する連絡先；

　　　　　　平林剛　（帝京大学溝口病院麻酔科　講師）電話：044-844-3333内線8233

3）試料の保管及び廃棄の方法

　　該当なし。

1. 研究に参加することにより起こりうる危険並びに必然的に伴う心身に対する不快な状態（有害事象）：

　本研究の麻酔は通常の麻酔で行う。VCV群の換気戦略は現在肺保護的とされる一般的な換気戦略である。PC-IRV群の換気戦略は一般的ではないが、換気効率が高く、換気量および気道内圧が軽減することが期待される。しかしauto PEEPによる肺過膨張が懸念される。肺過膨張が起こらないように、呼気フロー波形から適切な吸気開始するように随時I:E比を調節し、細心の注意を払う。本研究で行うPC-IRV群の呼気時間は1.5秒以上であり、肺過膨張のリスクはほとんどない。

　また、死腔量、二酸化炭素排出量を測定するボリュームカプノメトリは泉工医科工業株式会社が開発中の測定器だが、メインストリーム方式のガス・フローセンサーを気管チューブと呼吸回路の間に挿入するのみで測定できるため患者に害を及ぼすものではない。また、本研究により手術操作を妨げることはない。橈骨動脈カテーテルは通常の診療上必要な症例が対象となるため、本研究の実施のために加わる手技ではない。採血量は合計数mlを数回程度と考えられる。したがって本研究による参加者個人への利益、不利益はないと考える。

　研究によって起こりうる有害事象としては過度の高気道内圧、低換気、低酸素血症、高二酸化炭素血症、低血圧などが起こり得る。そのような場合は研究を速やかに中止し、治療とともに最善の方法に切り替える。ただし、本研究で行われる呼吸設定は、肺保護的と考えられている手段であり、患者の危険性は低いと思われる。

1. 研究に参加することにより生じる利益および試験期間中にかかる対象者の医療費：

　各種サイトカイン、IL-1β、 IL-6、IL-8、TNFαの測定費用は麻酔科研究費から支払われる。その他の麻酔、手術費用は保険診療の範囲内である。

1. 研究の中止の条件とその対応：

　患者に対し容認できない重篤な合併症、肺過膨張による気胸、肺障害、循環虚脱などを引き起こすことが明らかになった場合は研究を中止し、速やかに研究参加者および倫理委員会に報告する。中止時の情報・試料は5年間保管の後に廃棄する。重篤な合併症の判断は平林　剛（帝京大学溝口病院麻酔科　講師）および安藤富男（帝京大学溝口病院麻酔科　教授）が行う。

1. モニタリング及び監査：

モニタリング及び監査は行わない。

1. 研究実施後の研究対象者への対応：

　　被験者は研究終了後においても研究の結果によって得られた最善の予防、診断および治療を受けることができるように努める。

1. 研究成果の取りまとめとその扱い：

　個人情報匿名化した上で学術集会、医学雑誌への発表の可能性あり。

1. 研究資金源：

　特に費用は発生しない。

1. 利益相反：

　ボリュームカプノメトリは泉工医科工業株式会社から無償提供される。研究に関わる費用については医局研究費でまかない、泉工医科工業株式会社が負担することはない。本研究の測定については、客観的指標を用い、恣意的な判断が入らないようにする。データの解析については平林　剛（帝京大学溝口病院麻酔科　講師）の責任で公正に行うため、データの解析を泉工医科工業株式会社が行うことはない。

1. 倫理委員会への経過および最終報告予定：

　研究開始時期，実施状況（症例数）、倫理的配慮の状況、不利益・有害事象の発生状況、研究成果などについて、研究の終了時に中間報告書および最終報告書を倫理委員会に提出する。

1. 添付資料一覧：

なし。

1) Kilpatrick B, Slinger P. Lung protective strategies in anaesthesia. British Journal of Anaesthesia 2010. 105 (S1): i108–i116.

2) Roze H, Lafargue M, Batoz H, Picat MQ, Perez P, Ouattara A, Janvier G. Pressure-controlled ventilation and intrabronchial pressure during one-lung ventilation. British Journal of Anaesthesia 2010, 105 (3): 377–81.

3) Lee SM, Kim WH, Ahn HJ,3 J. A. Kim, Yang MK, Lee CH, Lee JH, Kim YR, Choi JW. The effects of prolonged inspiratory time during one-lungventilation: a randomised controlled trial. Anaesthesia 2013, 68, 908–916.
