# Supplementary material for: Lung-protective properties of expiratory flow-initiated pressure-controlled inverse ratio ventilation: A randomised controlled trial
Source: PLoS One. 2020 Dec 17;15(12):e0243971. doi: 10.1371/journal.pone.0243971 (PMC7746151; doi:10.1371/journal.pone.0243971)
Supplement: S2 File — (DOCX) [file pone.0243971.s003.docx]

**Title: Lung-protective properties of expiratory flow-initiated pressure-controlled inverse ratio ventilation: A randomised controlled trial**

**English protocol**

1. **Background:**

Pressure-controlled inverse inspiratory to expiratory ratio ventilation has been used in acute respiratory distress syndrome. However, its clinical utility remains controversial. Earlier studies used pressure-controlled inverse ratio ventilation (PC-IRV) with an inspiratory to expiratory (I:E) ratio of 2:1 to 4:1 without individual adjustment; this resulted in a very short expiratory phase and increased the risk lung hyperinflation and circulatory depression. PC-IRV with an IE ratio that is individually adjusted by observing the expiratory flow-time wave can appropriately maintain moderate total positive end-expiratory pressure (total PEEP). We previously reported that such expiratory flow (EF)-initiated PC-IRV reduced physiological dead space (VD_phys_) without lung hyperinflation and circulatory depression in patients undergoing robot-assisted laparoscopic radical prostatectomy. We hypothesised that EF-initiated PC-IRV may have lung-protective properties because this method can lower mechanical stress on the lung tissue by reducing VD_phys_ and the ventilatory volume required to maintain the partial pressure of CO_2_ (*P*aCO_2_). This study primarily aimed to evaluate the lung-protective properties of EF-initiated PC-IRV and to compare the differences in the lung-protective properties between EF-initiated PC-IRV and volume-controlled ventilation (VCV) in patients undergoing robot-assisted laparoscopic radical prostatectomy. The secondary aim was to compare the ventilatory efficacy of these methods.

1. **Methods:**

**[Design]**

Interventional, prospective, mono-center, single-blinded, randomized controlled clinical trial.

**[Locations]**

Mizonokuchi Hospital Teikyo University School of Medicine, Kanagawa, Japan

**[Subjects]**

Inclusion Criteria: 18 to 85 years with American Society of Anesthesiologists (ASA) physical status I or II and who were scheduled for robot-assisted laparoscopic radical prostatectomy.

Exclusion criteria: ASA physical status 3 to 5, a history of pneumothorax, and previous lung surgery.

**[Randomization and blinding]**

After written informed consent was obtained from eligible patients, researchers at the Teikyo Academic Research Centre randomised patients to the VCV or EF-initiated PC-IRV groups with a 1:1 allocation ratio using an envelope method after generating the allocation sequence.

Only the patient remained blinded during the whole study procedure.

**[Interventions and ventilatory strategies]**

No. of arms; 2 (VCV group and EF-initiated PC-IRV group)

Purpose of intervention; Treatment

Type of intervention; Maneuver

Ventilator settings were switched to the EF-initiated PC-IRV or VCV strategy following the establishment of the 25–30° Trendelenburg position and CO_2_ pneumoperitoneum at 12 mmHg.

The EF-initiated PC-IRV strategy included the pressure-controlled ventilation-volume guaranteed (PCV-VG) mode. In this mode, the airway pressure is adjusted to achieve a target tidal volume, and plateau pressures are allowed to rise to an upper limit of 30 cmH_2_O. BAP was set off. The initial respiratory rate was 12 beats min^-1^. The I:E ratio was individually adjusted by observing the expiratory flow-time wave. I:E ratios of 2:1, 1.5:1, or 1:1 were selected so that inspiration was initiated at the midpoint between the expiratory flow change point and the return point to the expected baseline.

The VCV strategy included the VC mode and a pause ratio of 20% was used in order to measure plateau pressure. A target tidal volume was set with plateau pressures upper limit of 30 cmH_2_O. BAP was set to 5 cmH_2_O. The I:E ratio was 1:2, and the initial respiratory rate was 12 beats min^-1^. Both strategies allowed for an increase in the respiratory rate to an upper limit of 18 beats min^-1^ to achieve a PaCO_2_ of less than 50 mmHg that was estimated from end-tidal CO_2_ (E_T_CO_2_) changes and differences between E_T_CO_2_ and PaCO_2_ on arterial blood gas analysis. Hypercapnia (> 50 mmHg) was permitted if the respiratory rate increased to 18 beats min^-1^ with a plateau pressure of 30 cmH_2_O.

Haemodynamics were maintained throughout the study with a mean arterial pressure (MAP) >70 mmHg, CI >2 l min^-1^ m^-2^, and an SVV <15%. If MAP fell below 70 mmHg, intravenous ephedrine (4–8 mg) was administered. If the SVV exceeded 15%, an additional intravenous fluid challenge was provided with 10 ml kg^-1^ of Ringer’s acetate solution or hydroxyethyl starch. Pulse oximetry-monitored oxygen saturation was allowed to drop to a lower limit of 93%. When these parameters exceeded the predetermined limits, alveolar recruitment manoeuvre consisting of applying a continuous positive airway pressure of 30 cmH_2_O for 30 seconds were conducted and the ventilator setting was changed by increasing the respiratory rate and FiO_2_ and increasing or decreasing the set tidal volume.

1. **Outcomes.**

The primary outcome was the change in serum IL-6 levels, which was used as a surrogate marker for both surgical- and ventilator-induced lung injury. The proinflammatory cytokines, IL-8 and IL-1β, were also evaluated. Each cytokines were measured at T_Baseline_ (20 min after the initial setting) and T_End_ (end of surgery).

The secondary outcome included VD_phys_, which was calculated as [VD_phys_ = V_TE_ × (PaCO_2_ - P_E_CO_2_) PaCO_2_^-1^]. Expired tidal volume (V_TE_) and expired CO_2_ partial pressure (P_E_CO_2_) were measured with volumetric capnography. Static compliance (C_stat_) was modified as [C_stat_ = V_TI_/(P_plat_ - PEEP)] (V_TI_, inspired tidal volume measured by volumetric capnography; P_plat_, plateau airway pressure). Driving pressure was calculated as (Driving pressure = P_plat_ – PEEP). Each respiratory or haemodynamic parameters were measured at T_Baseline_ and T_2h_ (2 h after intervention). The incidence of permissive hypercapnia (PaCO_2_ > 50 mmHg) and respiratory complications were also recorded.

1. **Statistical analysis**

A power analysis (α=0.05, β=0.2) indicated that at least 28 patients were required to detect differences in serum cytokine levels between the two strategies. The Mann–Whitney U test is used to compare differences of the serum cytokine levels between the two groups. Respiratory and haemodynamic continuous variables are analysed with Student’s t-test. The association between serum cytokines and other parameters is evaluated using Spearman's correlation coefficient, and the association between the other continuous variables is evaluated using a linear regression analysis. P-values <0.05 were considered statistically significant.

1. **Observation period:**

All data is measured during anaesthesia period.

1. **Project group/investigators:**

Department of Anaesthesiology

Mizonokuchi Hospital Teikyo University School of Medicine, Kanagawa, Japan

Go Hirabayashi; project leader

Naoko Kamosida, Hiroko Nishioka, Yuki Akihisa, Koichi Maruyama, and Tomio Andoh.

1. **Funding:**

The author(s) received no specific funding for this work.

1. **References:**

1) Kilpatrick B, Slinger P. Lung protective strategies in anaesthesia. British Journal of Anaesthesia 2010. 105 (S1): i108–i116.

2) Roze H, Lafargue M, Batoz H, Picat MQ, Perez P, Ouattara A, Janvier G. Pressure-controlled ventilation and intrabronchial pressure during one-lung ventilation. British Journal of Anaesthesia 2010, 105 (3): 377–81.

3) Lee SM, Kim WH, Ahn HJ,3 J. A. Kim, Yang MK, Lee CH, Lee JH, Kim YR, Choi JW. The effects of prolonged inspiratory time during one-lungventilation: a randomised controlled trial. Anaesthesia 2013, 68, 908–916.
