# Supplementary material for: Lung-protective properties of expiratory flow-initiated pressure-controlled inverse ratio ventilation: A randomised controlled trial
Source: PLoS One. 2020 Dec 17;15(12):e0243971. doi: 10.1371/journal.pone.0243971 (PMC7746151; doi:10.1371/journal.pone.0243971)
Supplement: Attachment — Submitted filename: revision suggestions.docx [file pone.0243971.s005.docx]

It is well written and well designed study

These are my suggestions

It would be better to give the ventilator strategy as a scheme in to the method part

It would be better to add VDshunt/VTE , ETCO_2 ,_ PaCO_2_-PetCO_2_  in results and table 2

Is PVC statement in line 306 actually PCV ?
