# Supplementary material for: Lung-protective properties of expiratory flow-initiated pressure-controlled inverse ratio ventilation: A randomised controlled trial
Source: PLoS One. 2020 Dec 17;15(12):e0243971. doi: 10.1371/journal.pone.0243971 (PMC7746151; doi:10.1371/journal.pone.0243971)
Supplement: Attachment — Submitted filename: revision_suggestions8.26.docx [file pone.0243971.s006.docx]

**Manuscript ID: PONE-D-20-10447**

**Title: Lung-protective properties of expiratory flow-initiated pressure-controlled inverse ratio ventilation: A randomised controlled trial**

Point-by-Point Response

Please note that the changes made do not influence the content, conclusions, or framework of the paper. We have not listed below all minor changes made; however, these are indicated in the revised manuscript.

1) Thank you for indicating that the study was supported by departmental funding. Before we can proceed, however, we ask that you please clarify:

a) The name of the department and of the university supported the study

b) Whether the support was financial or material

c) The grant numbers and any other relevant details

**Response:**

a) Department of Anaesthesiology, Mizonokuchi Hospital Teikyo University School of Medicine, Kanagawa, Japan.
b) Supported financially.
c) No other funding or grants were received..

The following text has been added to the revised manuscript:

“This study was financially supported by internal funding from the Department of Anaesthesiology, Mizonokuchi Hospital Teikyo University School of Medicine, Kanagawa, Japan. The funders had no role in study design, data collection and analysis, decision to publish, or preparation of the manuscript. No author received a salary from the funders. The authors received no other funding for this work.”

1. Please ensure that your manuscript meets PLOS ONE's style requirements, including those for file naming. The PLOS ONE style templates can be found at https://journals.plos.org/plosone/s/file?id=wjVg/PLOSOne_formatting_sample_main_body.pdf and

https://journals.plos.org/plosone/s/file?id=ba62/PLOSOne_formatting_sample_title_authors_affiliations.pdf

**Response:** We have named the files accordingly.

2. During our internal evaluation, the in-house editorial staff noted that the clinical trial was registered at ClinicalTrials.gov

'It was approved by the Ethical Committee of Teikyo University School of Medicine, Tokyo, Japan on 12 September 2017 (No. 17-063) and was registered at ClinicalTrials.gov UMIN000029552' (line 82-84).

However, the trial registration provided in the manuscript is a UMIN Clinical Trials Registry number. At this time, we ask that you also provide the ClinicalTrias.gov trial registration number in your Methods section.

**Response:** The corresponding text has been revised as follows:

“It was approved by the Ethical Committee of Teikyo University School of Medicine, Tokyo, Japan on 12 September 2017 (No. 17-063), and was registered with the University Hospital Medical Information Network Clinical Trials Registry (UMIN000029552).”

3. In the Methods section, please confirm that the study protocol provided in Supplementary Figure 1 is the original study protocol that was specifically submitted to and approved by the Ethical Committee of Teikyo University School of Medicine.

**Response:** The corresponding text has been revised as follows:

“The original study protocol, in Japanese, approved by the Ethical Committee of Teikyo University School of Medicine is provided in Supporting Information File S1; an English translation is provided in in Supporting Information File S2.”

4. In your Methods section, please provide additional information about the participant recruitment method and the demographic details of your participants. Please ensure you have provided sufficient details to replicate the analyses such as: a) the recruitment date range (month and year).

**Response**: The following information has been added to the revised manuscript:

“Participants were recruited between December 2018 and September 2019.”

5. Please provide a sample size and power calculation in the Methods, or discuss the reasons for not performing one before study initiation.

**Response:** The relevant text has been revised as follows, and a reference [14] has been added:

“Based on previous study data [14], the calculated sample size for detecting differences in IL-6 concentration between ventilatory settings was 14 participants per group when using a one-sided test with a power of 0.8 and type I error rate of 0.05, to give an estimated difference of 1 of the parameter’s standard deviation (SD).”

14. Michelet P, D'Journo X-B, Roch A, Doddoli C, Marin V, Papazian L, et al. Protective ventilation influences systemic inflammation after esophagectomy: a randomized controlled study. Anesthesiology. 2006;105: 911-919.

6. Thank you for stating the following financial disclosure:

 [The funders had no role in study design, data collection and analysis, decision to publish, or preparation of the manuscript.].

At this time, please address the following queries:

a. Please clarify the sources of funding (financial or material support) for your study. List the grants or organizations that supported your study, including funding received from your institution.

**Response:** This study was supported by departmental funding.

b. State what role the funders took in the study. If the funders had no role in your study, please state: “The funders had no role in study design, data collection and analysis, decision to publish, or preparation of the manuscript.”

**Response:** The funders had no role in the study design, data collection and analysis, decision to publish, or preparation of the manuscript.

c. If any authors received a salary from any of your funders, please state which authors and which funders.

**Response:** No author received a salary from any of the study funders.

d. If you did not receive any funding for this study, please state: “The authors received no specific funding for this work.”

**Response:** The authors received no specific funding for this work.

Please include your amended statements within your cover letter; we will change the online submission form on your behalf.

**Response:** As instructed, the amended statements have been included within the cover letter.

[Note: HTML markup is below. Please do not edit.]

Reviewers' comments:

Reviewer's Responses to Questions

Comments to the Author

1. Is the manuscript technically sound, and do the data support the conclusions?

The manuscript must describe a technically sound piece of scientific research with data that supports the conclusions. Experiments must have been conducted rigorously, with appropriate controls, replication, and sample sizes. The conclusions must be drawn appropriately based on the data presented.

Reviewer #1: Partly

Reviewer #2: Yes

Reviewer #3: Partly

2. Has the statistical analysis been performed appropriately and rigorously?

Reviewer #1: I Don't Know

Reviewer #2: Yes

Reviewer #3: No

3. Have the authors made all data underlying the findings in their manuscript fully available?

The [PLOS Data policy](http://www.plosone.org/static/policies.action#sharing) requires authors to make all data underlying the findings described in their manuscript fully available without restriction, with rare exception (please refer to the Data Availability Statement in the manuscript PDF file). The data should be provided as part of the manuscript or its supporting information, or deposited to a public repository. For example, in addition to summary statistics, the data points behind means, medians and variance measures should be available. If there are restrictions on publicly sharing data—e.g. participant privacy or use of data from a third party—those must be specified.

Reviewer #1: Yes

Reviewer #2: Yes

Reviewer #3: Yes

4. Is the manuscript presented in an intelligible fashion and written in standard English?

PLOS ONE does not copyedit accepted manuscripts, so the language in submitted articles must be clear, correct, and unambiguous. Any typographical or grammatical errors should be corrected at revision, so please note any specific errors here.

Reviewer #1: No

Reviewer #2: Yes

Reviewer #3: Yes

5. Review Comments to the Author

Please use the space provided to explain your answers to the questions above. You may also include additional comments for the author, including concerns about dual publication, research ethics, or publication ethics. (Please upload your review as an attachment if it exceeds 20,000 characters)

Reviewer #1: It is thought that the results of previous studies suggesting that the serum cytokines selected in this study could was used as a surrogate marker for lung injury should be included in the introduction or discussion. In addition, I think it is necessary to consider why there were no differences of cytokines between the two groups although EF-initiated PC-IRV reduce dead space. The primary endpoint of this study was not change of respiratory mechanics but the change of serum cytokines.

**Response:** We believe that EF-initiated PC-IRV would be lung-protective in cases with a long duration of surgery for patients with low respiratory compliance, and if both ventilatory strategies are utilized with target PaCO_2_ <50 mmHg and PaO_2_ >100 mmHg, but not the same tidal volume or plateau pressure <30 cmH_2_O. However, the patients included in this study were normal %VC patients and those who underwent short duration surgery conducted at plateau pressure <30 cmH_2_O.

Regarding the cytokine level difference, we have added the following text to the revised manuscript:

“Michelet P et al. [14] also evaluated serum IL-6 and IL-8 levels to compare the lung-protective strategy and conventional ventilation in patients undergoing esophagectomy. Mean duration of surgery was 300 min, which was longer than that in our study, and serum IL-6 and IL-8 levels increased more than those in our study, with the differences being significant. Serum IL-6 and IL-8 levels did not increase in case of short duration of anaesthesia, surgery, and CO_2_ pneumoperitoneum.”

Page 4, 91-93. Has the same surgeon performed surgery on all enrolled patients?

**Response:** No. The same surgeon did not perform surgery on all enrolled patients.

Page 6, 158-160. Please add references about the cytokines selected in this study.

**Response:** The following text and reference has been added to the revised manuscript:

“Michelet P et al. [14] also evaluated serum IL-6 and IL-8 levels to compare the lung-protective strategy and conventional ventilation in patients undergoing esophagectomy. Mean duration of surgery was 300 min, which was longer than that in our study, and serum IL-6 and IL-8 levels increased more than those in our study, with the differences being significant. Serum IL-6 and IL-8 levels did not increase in case of short duration of anaesthesia, surgery, and CO_2_ pneumoperitoneum.”

Page 6, 172-175. Was there data regarding serum cytokine obtained from previous studies or preliminary study for calculating the sample size? Please describe the sample size calculation in more detail.

**Response:** The relevant text has been revised as follows, and reference [14] has also been added:

“Based on previous study data [14], the calculated sample size for detecting differences in IL-6 concentration between ventilatory settings was 14 participants per group when using a one-sided test with a power of 0.8 and type I error rate of 0.05, to give an estimated difference of 1 of the parameter’s standard deviation (SD).”

14. Michelet P, D'Journo X-B, Roch A, Doddoli C, Marin V, Papazian L, et al. Protective ventilation influences systemic inflammation after esophagectomy: a randomized controlled study. Anesthesiology. 2006;105: 911-919.

Page 6, 175-178. What are the criteria for using non-parametric and parametric methods differently depending on the type of variable? Did you perform a normality test?

**Response:** Yes. The normality of the data was assessed and the corresponding tests were used depending on the results.

Page 9, 231-233. In this study, correlation analysis was performed between IL-6 and the duration of surgery. Did your group conduct correlation analysis between cytokine and duration of the Trendelenburg position?

**Response:** Yes, a correlation analysis was performed between IL-6 and the duration of surgery and the duration of the Trendelenburg position. The results of both comparisons showed a significant positive correlation; this has been mentioned in the revised manuscript.

Page 10, 270-272. The meaning of the sentence regarding permissive hypercapnia is not clear. Can permissive hypercapnia in two patients observed only in VCV give special significance compared to PC-IRV?

**Response**: We apologize for the lack of clarity. We have revised the corresponding text as follows:

“For two patients in the VCV group, ventilation became difficult and permissive hypercapnia was required (*P*aCO_2_ over 50 mmHg with plateau pressure of 30 cmH_2_O, respiratory rate of 18 bpm).”

Page 11, 280-282. Isn't the duration of the Trendelenburg position and pneumoperitoneum more meaningful than simply duration of surgery?

**Response:** Thank you for your comment. Accordingly, we have revised the corresponding text to add some data as follows:

“In both groups, there was a significant positive correlation between serum IL-6 at T_End_ and the duration of surgery (EF-initiated PC-IRV group: r = 0.82, P < 0.001; VCV group: r = 0.85, P < 0.001; Fig 2), and the duration of the Trendelenburg position and pneumoperitoneum (EF-initiated PC-IRV group: r = 0.83, P < 0.001; VCV group: r = 0.88, P < 0.001).”

Page 11, 303-308. The meaning of this paragraph is unclear. It was noted that the peak inspiratory pressure was lower in the PCV group than that in the VCV in patients with laparoscopy and robot surgery. Why were there advantages in maintaining respiratory mechanics?

**Response:** PCV reduces dead space, especially in low respiratory compliance cases, and enables reduction of tidal volume and plateau pressure.

Reviewer #2: It is well written and well designed study
These are my suggestions
It would be better to give the ventilator strategy as a scheme in to the method part
It would be better to add VDshunt/VTE , ETCO2 , PaCO2-PetCO2 in results and table 2
Is PVC statement in line 306 actually PCV ?

**Response:** Thank you for your suggestions. Accordingly, we have added the following data in the form of Table 1:

**Table 1. Ventilator strategies**

|  | VCV strategy |  | EF-initiated PC-IRV strategy |
| --- | --- | --- | --- |
| Ventilator mode | Volume control with a pause ratio of 20% |  | Pressure-controlled ventilation-volume guaranteed |
| Inspiratory:Expiratory ratio | 1:2 |  | A ratio of 2:1, 1.5:1, or 1:1 was selected so that inspiration was initiated at the midpoint between the EF change point and the return point to the expected baseline |
| Baseline airway pressure (cmH_2_O) | 5 |  | Off |
| Respiratory rate | The initial respiratory rate was 12 beats min^-1^; an increase in the respiratory rate to an upper limit of 18 beats min^-1^ was allowed to achieve a PaCO_2_ of less than 50 mmHg | | |
| Target tidal volume | Adjusted to achieve a plateau pressure upper limit of 30 cmH_2_O | | |

EF-initiated PC-IRV, expiratory flow-initiated pressure-controlled inverse ratio ventilation; PaCO_2_, partial pressure of CO_2_; VCV, volume-controlled ventilation.

We have added the E_T_CO_2_ data in the Results section and in Table 3 of the revised manuscript. As for VD_shunt_/V_TE_ and PaCO_2_-PetCO_2_, we would like to describe these in a further study.

Thank you for pointing out the inadvertent error; accordingly, “PVC” was revised to “PCV”.

Reviewer #3: A prospective two-arm randomized clinical trial was conducted to compare differences in serum cytokine levels in men undergoing robot-assisted laparoscopic radical prostatectomy. No significant differences in IL-6 levels at the end of surgery were observed between the two arms.

Minor revisions:
1- Line 91: Provide specific details on how the randomization list was generated. If randomized blocks were used, indicate the block size.

**Response:** The randomisation list was generated by researchers at Teikyo Academic Research Centre.

2- Line 172: Provide more complete details for the statistical power calculation. The power calculation should include: sample size, alpha level (indicating one or two-sided), minimal detectable difference and statistical testing method.

**Response:** The relevant text has been revised as follows, and reference [14] has also been added:

“Based on previous study data [14], the calculated sample size for detecting differences in IL-6 concentration between ventilatory settings was 14 participants per group when using a one-sided test with a power of 0.8 and type I error rate of 0.05, to give an estimated difference of 1 of the parameter’s standard deviation (SD).”

14. Michelet P, D'Journo X-B, Roch A, Doddoli C, Marin V, Papazian L, et al. Protective ventilation influences systemic inflammation after esophagectomy: a randomized controlled study. Anesthesiology. 2006;105: 911-919.

3- Line 175 states, “The Mann–Whitney U test was used to compare differences in the serum
cytokine levels between the two groups.” To clarify, was the Mann-Whitney U test used to compare differences only at the end of surgery?

**Response:** The test was used at baseline and the end of surgery.

4- Line 187: Clarify that the patients were randomized rather than simply divided.

**Response:** The corresponding text has been revised as follows:

“…28 eligible patients who were randomly divided into the following two groups: …”.

5- Table 1: In the statistical analysis section, state the statistical methods used to compare patient and surgery characteristics shown in Table 1. If data is normally distributed, summarize the results using mean and standard deviation. If the data is not normally distributed provide the median, first and third quartiles. Use nonparametric tests (Mann-Whitney U-test) to compare groups when the distribution of the data is not normally distributed. Use parametric tests (t-tests) to compare groups with normally distributed data.

**Response:** The corresponding text has been revised as follows:

“For the normally distributed respiratory and haemodynamic continuous variables, data were analysed using Student’s t-test. The Mann–Whitney U test was used to compare groups when the distribution of the data was not normally distributed.”

6- Table 2: Tests of the interaction of arm by time is more appropriate than repeatedly applying t-tests when comparing the data shown in Table 2.

**Response:** At baseline, the VCV strategy was used in both groups, same arm. We think that tests of the interaction of arm by time may not be appropriate.

7- Line 232: Clarify the rs notation. There appears to be a typographical error “rs=0,082”.

**Response:** Thank you for noting this inadvertent error, which we have revised as follows:

“r = 0,082”

8- The p-value associated with a correlation is a test of the null hypothesis: correlation equal to zero; however, the absolute magnitude of the coefficient indicates the strength of the linear relationship between two variables. In general, the strength or correlation coefficient is the more important statistic to reflect upon.
Below is a table for interpreting correlation coefficients:
Coefficient (absolute value) Interpretation
0.90 - 1.0 Very Strong
0.70 - 0.89 Strong
0.40 - 0.69 Moderate
0.10 - 0.39 Weak
less than 0.10 Negligible correlation

**Response:** Thank you for this clarification.

9- Indicate if adverse events were collected according to a standardized method.

**Response:** As has been mentioned in the Results section, no instances of respiratory complications were recorded.

10- Add the correlation coefficients to Figures 2 and 3.

**Response:** The correlation coefficients have been added to Figures 2 and 3.

11- Indicate the funding source(s), and the role of the funder(s)?

**Response:** The relevant information has been added to the revised manuscript:

“Funding

This study was supported by department funding.”

12- All acronyms and abbreviations must be spelled out in first use.

**Response**: Thank you. We have checked the text to ensure that this guideline is followed.

6. PLOS authors have the option to publish the peer review history of their article ([what does this mean?](https://journals.plos.org/plosone/s/editorial-and-peer-review-process#loc-peer-review-history)). If published, this will include your full peer review and any attached files.

If you choose “no”, your identity will remain anonymous but your review may still be made public.

Do you want your identity to be public for this peer review? For information about this choice, including consent withdrawal, please see our [Privacy Policy](https://www.plos.org/privacy-policy).

Reviewer #1: No

Reviewer #2: No

Reviewer #3: No

[NOTE: If reviewer comments were submitted as an attachment file, they will be attached to this email and accessible via the submission site. Please log into your account, locate the manuscript record, and check for the action link "View Attachments". If this link does not appear, there are no attachment files.]

While revising your submission, please upload your figure files to the Preflight Analysis and Conversion Engine (PACE) digital diagnostic tool, <https://pacev2.apexcovantage.com/>. PACE helps ensure that figures meet PLOS requirements. To use PACE, you must first register as a user. Registration is free. Then, login and navigate to the UPLOAD tab, where you will find detailed instructions on how to use the tool. If you encounter any issues or have any questions when using PACE, please email PLOS at [figures@plos.org](mailto:figures@plos.org). Please note that Supporting Information files do not need this step.

*In compliance with data protection regulations, you may request that we remove your personal registration details at any time.* [*(Remove my information/details)*](https://www.editorialmanager.com/pone/login.asp?a=r)*. Please contact the publication office if you have any questions.*
