# Supplementary material for: Lung-protective properties of expiratory flow-initiated pressure-controlled inverse ratio ventilation: A randomised controlled trial
Source: PLoS One. 2020 Dec 17;15(12):e0243971. doi: 10.1371/journal.pone.0243971 (PMC7746151; doi:10.1371/journal.pone.0243971)
Supplement: Attachment — Submitted filename: revision_suggestions_10_26.docx [file pone.0243971.s007.docx]

**Manuscript ID: PONE-D-20-10447**

**Title: Lung-protective properties of expiratory flow-initiated pressure-controlled inverse ratio ventilation: A randomised controlled trial**

**Point-by-Point Responses**

**Comments from Reviewer 3:**

If data is normally distributed, summarize using mean (SD) and compare using parametric methods, possibly t-tests. However, if data is non-parametic summarize using median (first and third quartiles) and compare using non-parametic method such as Mann Whitney U tests.

**Response:**

Thank you for your comment. We have summarized our parametric data using mean (SD) and our non-parametric data using median (first and third quartiles) in the revised manuscript. We have also ensured that the parametric data comparisons were performed using Student’s t-test and that those of the non-parametric data analyses were conducted using the Mann-Whitney U test. This information has also been added to the revised manuscript.

“All parametric data were described using mean (SD) and the non-parametric data using median (first and third quartiles). Parametric data comparisons were performed using Student’s t-test and non-parametric data analyses were conducted using the Mann-Whitney U test.”

We also revised Table 4 accordingly.

In addition, Table 4 was revised from:

**Table 4. Changes in serum cytokine levels**

|  | **Time period** | **VCV group** | **EF-initiated PC-IRV group** | **^a^P-value** |
| --- | --- | --- | --- | --- |
| IL-6 (pg ml^-1^) | T_Baseline_ | 8 ( 8-8 [8-8]) | 8 ( 8-8 [8-8]) | - |
|  | T_End_ | 31 (24.5-59 [18-273]) | 34 (20.5-63.5 [10-122]) | 0.84 |
| IL-8 (pg ml^-1^) | T_Baseline_ | 8 ( 8-8 [8-8]) | 8 ( 8-8 [8-8]) | - |
|  | T_End_ | 8 (8-9 [8-21]) | 8 (8-9.75 [8-121]) | 0.62 |

Values are presented as the median [IQR]. ^a^P-values are from The Mann–Whitney U test. VCV, volume-controlled ventilation; EF-initiated PC-IRV, expiratory flow-initiated pressure-controlled inverse ratio ventilation; T_Baseline_, 20 min after the initial setting; T_End_, end of surgery.

To:

**Table 4. Changes in serum cytokine levels**

|  | **Time period** | **VCV group** | **EF-initiated PC-IRV group** | **^a^P-value** |
| --- | --- | --- | --- | --- |
| IL-6 (pg ml^-1^) | T_Baseline_ | 8 [8-8] | 8 [8-8] | - |
|  | T_End_ | 31 [24.5-59] | 34 [20.5-63.5] | 0.84 |
| IL-8 (pg ml^-1^) | T_Baseline_ | 8 [8-8] | 8 [8-8] | - |
|  | T_End_ | 8 [8-9] | 8 [8-9.75] | 0.62 |

Values are presented as the median [first and third quartiles]. ^a^P-values are from The Mann–Whitney U test. VCV, volume-controlled ventilation; EF-initiated PC-IRV, expiratory flow-initiated pressure-controlled inverse ratio ventilation; T_Baseline_, 20 min after the initial setting; T_End_, end of surgery.
